# An analysis of the usefulness for using skin adhesive without closed-suction drainage in primary total hip arthroplasty: A retrospective propensity score matched study

**DOI:** 10.1097/MD.0000000000035181

**Published:** 2023-09-15

**Authors:** Chan Young Lee, Sheng-Yu Jin, Hwiwoo Jeong, Taek-Rim Yoon, Kyung-Soon Park

**Affiliations:** a Department of Orthopedic Surgery, Chonnam National University Medical School and Hwasun Hospital, Hwasun-gun, Republic of Korea

**Keywords:** closed suction surgical system, complications, skin adhesives, total hip arthroplasty

## Abstract

The closed suction surgical drainage system (CSSD) is routinely used after total hip arthroplasty (THA) by orthopedic surgeons in many institutions. However, it has not been shown to decrease the rate of wound infection significantly and may even increase blood loss. This study aimed to evaluate the usefulness of using skin adhesive without CSSD in uncomplicated THA. From July 2015 to September 2017, 200 patients undergoing unilateral THA were enrolled and divided into 2 groups, either receive CSSD (134 patients) or not receive CSSD (66 patients). Then, the propensity matched was performed. Calculated total blood loss, changes in hemoglobin (Hgb) level, transfusions were evaluated. In addition, data on the length of hospital stay, operation time, closure time, time to using crutches following THA were collected. Finally, Harris hip score (HHS), total estimated cost, and complications were assessed. The non-CSSD group had comparatively less blood loss (508.5 ± 280.3 mL compared with 742.1 ± 330.3 mL, *P* < .001), fewer transfusions (0.03 units compared with 0.3 units, *P* = .02), less transfusion rate (1.9% compared with 17.3 %, *P* = .02), lower change of Hgb from immediate postoperative period to 3 days later(1.6 ± 1.0 g/dL compared with 2.0 ± 0.8 g/dL, *P* = .03), than the CSSD group. There was a longer duration of hospital stay in the CSSD groups (7.2 days compared with 7.8 days, *P* = .03) The mean total cost in the non-CSSD group was $162.1, which was less than that of the CSSD group, which spent $288.5 on average (*P* < .001). there was 1 allergic reaction in the non-CSSD group (*P* = .32). The use of skin adhesive without CSSD could help decrease blood loss, the need for transfusion, and the length of hospital stay, and seems to more cost-effectiveness than using CSSD. It may also provide superior results and allow the patient to recover faster. Using this type of skin adhesive without CSSD is an efficient wound closure method for patients undergoing uncomplicated THA. However, care must be taken for allergic reactions, especially for patients with known or suspected allergies to cyanoacrylate or formaldehyde.

## 1. Introduction

Total hip arthroplasty (THA) surgery has proved to be a successful and cost-efficient procedure in the field of orthopedics today. There has been a focus on improving procedure-related issues, including postoperative infection and wound closure techniques.

The use of the closed suction surgical drainage system (CSSD) after surgery is a routine practice in many institutions, and a CSSD has been commonly used following THA. In the past, many surgeons believe that the use of CSSD may reduce the risk of hematoma formation and infection. However, many recent studies indicate that the routine use of drainage has no benefit.^[1]^ Xu et al found that drain use was associated with a higher transfusion rate and a longer postoperative length of stay.^[2]^ Willett et al indicated that the drain continued presence resulted in minimal further drainage and did not reduce the likelihood of hematoma formation in patients undergoing routine primary.^[3]^ Drainage may increase the need for transfusion due to increased blood loss after total joint replacement.^[4]^ In response to the arguments mentioned above, a new strategy of closed suction drainage after primary THA was introduced: closed suction drainage under the influence of gravity in the first 24 hours after THA.^[5]^ The new method could help resolve some of the issues, such as those involving blood and transfusion, that were previously associated with later complications of the traditional closed suction drainage system.

The commonly used wound closure methods after orthopedic surgery include skin staples and sutures. A new generation of wound closure techniques has been developed, with the advent of new kinds of skin adhesives. 2-Octyl cyanoacrylate—also known as Dermabond (Ethicon Inc, Somerville, NJ)—has been commonly used in wound closure as a tissue adhesive since 1949.^[6]^ Currently, the FDA had approved the skin adhesive application for open wounds in humans. Skin adhesive is a superior alternative to surgical staples in the high-tension surgical wound environments of total joint arthroplasty incisions.^[7]^ Skin adhesive could create an immediate watertight seal in a sterile operative environment and provide a barrier to microorganisms.^[8]^ However, to our knowledge, there have been few studies to date comparing wounds with CSSD and wounds without CSSD for patients with uncomplicated THA.

The primary objective of this study was to assess the effects of using skin adhesive without CSSD on calculated total blood loss, change in hemoglobin (Hgb), cost-effectiveness (total estimated cost, length of hospital stay), and complications. The secondary objective was to measure operation time including wound closure time, and functional outcomes (time to start ambulation, Harris hip score [HHS]).

## 2. Methods

### 2.1. Patients

**Figure 1.**
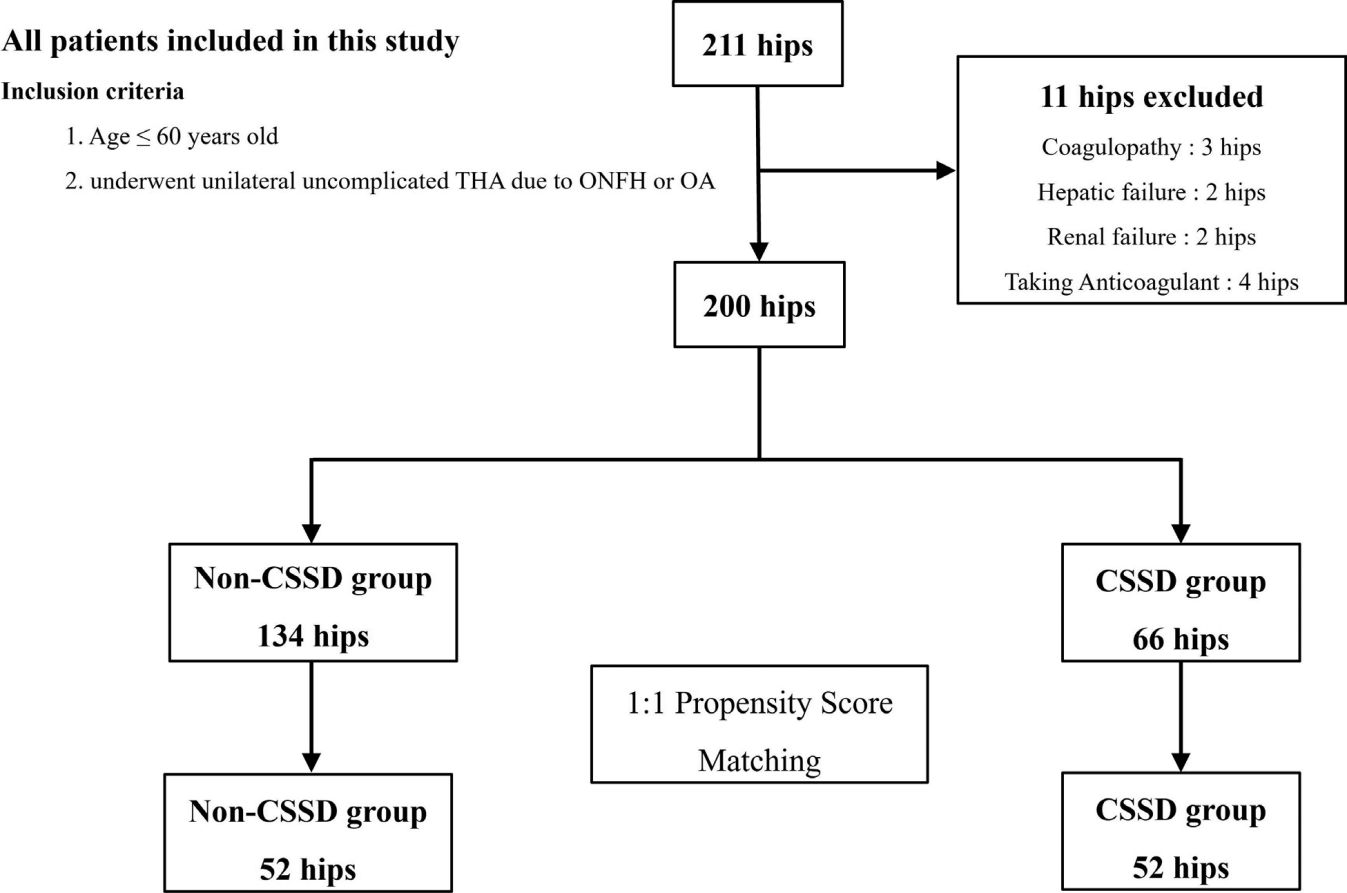
Flowchart of propensity score matching.

The study was approved by the Institutional Review Board of Chonnam National University Hwasun Hospital (IRB No. CNUHH-2020-183). We included patients who were 60 years old or younger and were admitted to Chonnam National University Hwasun Hospital for a unilateral uncomplicated THA due to osteonecrosis of the femoral head or osteoarthritis of the hip joint from July 2015 to September 2017. We excluded 9 patients with coagulopathies (hematologic disease, hepatic failure, and renal failure) and those requiring regular anticoagulation due to preexisting medical conditions such as cardiac disease and other medical co-morbidities. A total of 200 patients were enrolled in this study after applying the inclusion and exclusion criteria (Fig. 1).

### 2.2. Matching

Among the 200 patients, 134 received Steri-Strips (3M, Neuss, Germany) with CSSD, and 66 received skin adhesives without CSSD. They were 1:1 propensity score-matched using greedy nearest-neighbor matching algorithm without replacement.^[9]^ The propensity scores were generated from a logistic regression with the use of age, sex, body mass index, American Society of Anesthesiologists physical status classification system, preoperative HHS, and Hgb level 2 weeks preoperatively. The caliper distance was set as 0.2 standardized deviations of the logit of the propensity scores. After propensity-matched analysis, 104 patients were finally included in this study who underwent THA with or without CSSD, and each cohort had 52 cases (Table 1, Fig. 1).

**Table 1:** Patient demographic data.

	Unmatched				After propensity matched			
	non-CSSD (N = 66)	CSSD (N = 134)	*P* value	SMD	non-CSSD (N = 52)	CSSD (N = 52)	*P* value	SMD
Age (Yr)	45 ± 10.2	43 ± 9.7	.35	0.20	45 ± 9.8	45 ± 9.2	>.99	<0.001
Gender (male/female)	27/39	77/57	**.04**	0.37	26/26	24/28	.85	0.09
BMI (kg/m^2^)	24.0 ± 3.3	24.7 ± 4.1	.30	0.18	24.2 ± 3.3	23. 8 ± 3.7	.45	0.11
ASA	2.1 ± 0.5	1.9 ± 0.6	**.03**	0.35	1.9 ± 0.5	1.9 ± 0.5	.35	<0.001
HHS (preoperative)	44.3 ± 12.1	44.5 ± 10.6	.90	0.02	44.8 ± 12.0	45.8 ± 9.6	.65	0.09
Hgb level (2 wk preoperative)	13.0 ± 1.4	12.8 ± 1.2	.26	0.16	13.3 ± 1.4	12.9 ± 1.3	.20	0.30
Diagnosis								
ONFH/OA	40/26	52/82	**.004**	0.49	26/26	22/30	.43	0.17
Acetabular components			.61	0.08			.43	0.17
Bencox (Corentec, Seoul, Korea)	36	68			27	23		
Delta PF (Lima, Udine, Italy)	30	66			25	29		
Femoral components			.61	0.08			.43	0.17
Bencox ID (Corentec, Seoul, Korea)	36	68			27	23		
M/L taper (Zimmer, Warsaw, USA)	30	66			25	29		

ASA = American society of anesthesiologists physical status classification system, BMI = body mass index, CSSD = closed suction surgical drainage system, HHS = Harris hip score, Hgb = hemoglobin, OA = osteoarthritis, ONFH = osteonecrosis of the femoral head, SMD = standardized mean difference.

### 2.3. Drainage system & skin closure

**Figure 2.**
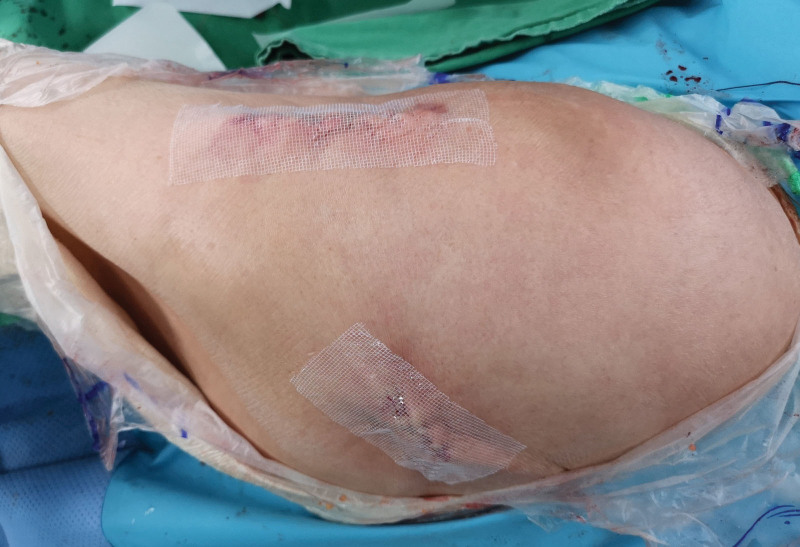
Intraoperative views of wound closure using skin adhesive in the non-CSSD group. CSSD = closed suction surgical drainage system.

**Figure 3.**
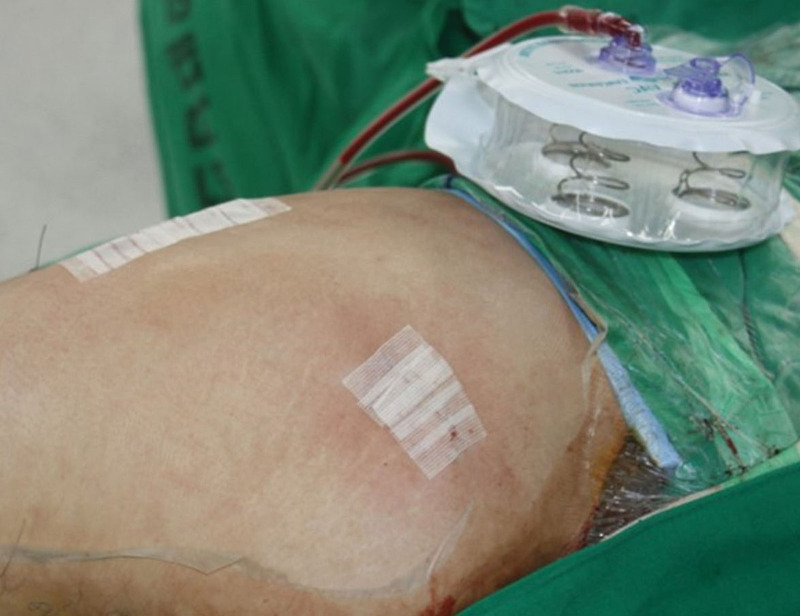
Intraoperative views of the closed suction surgical drainage (CSSD) group.

THA was performed using minimally invasive 2 incision approach by a experienced orthopedic surgeon.^[10]^ They used cementless acetabular cup and cementless femoral stem. After the implants were placed, the anterior capsule and fascia were sutured with number 1 VICRYL polyglactin 910 (Ethicon, NJ). Subcuticular closure was performed in layers with 1-0 VICRYL and an antibacterial suture (Ethicon, NJ). For the non-CSSD group, skin adhesive with a flexible, self-adhesive polyester mesh (Dermabond Prineo, Ethicon, NJ) was first applied, and then a padded Tegaderm (3M, Saint Paul, MN) dressing was added for observation (Fig. 2). For the CSSD group, 1 drainage tube was placed in the joint and subsequently brought out through the skin. The patients had Steri-Strips^TM^ applied for wound closure, and their dressings were changed daily with standard gauze (Fig. 3). The closed suction system was maintained until the amount of drained blood per day was <100 mL. The drained blood was measured every morning at 6 o’clock, and if it was confirmed to be <100 mL, it was removed on the same day. Postoperatively, intermittent pneumatic compression prevented venous thromboembolism.

### 2.4. Data assessment

Intraoperative blood loss was calculated from drain volumes and swab weights. Hgb levels were measured at 2 weeks before surgery and on days 1 and 3 postoperatively. The drop in the Hgb level was then calculated. If the Hgb level was <8.0 g/dL, a transfusion of packed red cells was performed. The Hgb level was checked from the surgical stages of incision to complete wound closure. The outcomes of calculated total blood loss were obtained using modified equations in the literature.^[11]^


$$Calculated\;total\;blood\;loss\;\left(mL\right)=1000*\frac{Preoeprative\;Hgb-\;POD\;3\;days\;Hgb\;}{preoperative\;Hgb}+\;Transfusion\;amount$$

The numbers of total transfusions used intraoperatively and postoperatively were recorded. The operation time was determined from the point when the incision was made to wound closure. Total wound closure time was defined as the time taken for the subcutaneous tissues and skin layers to close.

The changes in Hgb level, transfusion amount, and transfusion rate were used as the primary outcome measures. The secondary outcomes were the length of hospital stay, blood loss, operation time, closure time, HHS total estimated cost, and complications.

The wound dehiscence, superficial or wound infection, hematoma formation during the hospitalization period, and deep infection during the follow-up period were recorded. We checked the HHS and radiographs at each follow-up.^[12]^ We observed complications such as infection, periprosthetic fracture, dislocation, aseptic loosening, and heterotopic ossification. All the patients were followed up at 1 month, 3 months, 6 months, 1 year, and annually thereafter.

### 2.5. Statistical analysis

The independent samples t-test between both groups was performed for the analysis of operation time, closure time, calculated total blood loss, transfusion amount, postoperative HHS, Hgb level, changes in Hgb, and total estimated cost between groups. The rates of transfusion were compared using Fisher exact test. All statistical comparisons were done using SPSS ver. 25.0 software (SPSS, Chicago, IL). A *P* value < .05 was considered statistically significant.

## 3. Results

The non-CSSD group had comparatively less blood loss (508.5 ± 280.3 mL compared with 742.1 ± 330.3 mL, *P* < .001), fewer transfusions (0.03 units compared with 0.3 units, *P* = .02), less transfusion rate (1.9% compared with 17.3 %, *P* = .02) than the CSSD group. Also, the change of Hgb from immediate postoperative period to 3 days later was lesser in the non-CSSD group (1.6 ± 1.0 g/dL compared with 2.0 ± 0.8 g/dL, *P* = .03). The results for the Hgb levels exclude patients who received transfusions. There was a longer duration of hospital stay in the CSSD groups (7.2 days compared with 7.8 days, *P* = .03). There was a significant difference in total wound closure time between groups. The CSSD group took 12.8 minutes, while the non-CSSD group took 14.2 minutes for would closure (*P* < .001). For functional outcomes, there was no significant difference in mean HHS between both groups preoperatively, 1 and 3 months postoperatively, and at the last follow-up. However, the mean time to using crutches following THA in the CSSD group was longer than that in the non-CSSD group (1.8 ± 0.8 days compared with 2.6 ± 1.4 days, *P* = .002). The mean total cost in the non-CSSD group was $162.1, which was less than that of the CSSD group, which spent $288.5 on average (*P* < .001). The primary costs in the CSSD group were the drainage fees, nursing care fees, the daily dressing changes, and transfusions, whereas in the non-CSSD group, it was the cost of the skin adhesive.

**Figure 4.**
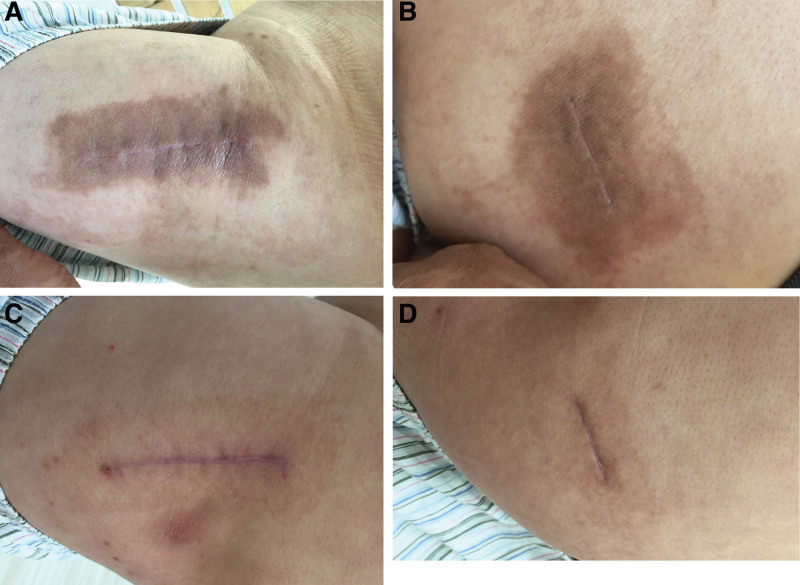
A 58-yr-old man represented pigmented contact dermatitis just after the first use of skin adhesive for the wound. (A) Hyperpigmentation observed at the anterior incision area with no signs of dermatitis at 1 mo after THA, and (B) at the posterior incision. (C) The local allergic reaction had resolved 3 mo later at the anterior incision area, and (D) at the posterior incision. THA = total hip arthroplasty.

There were no reported cases of superficial or wound infections, wound dehiscence, and hematoma in both groups. However, 1 58-year-old man developed an allergic skin reaction to skin adhesive 1 month after surgery in the non-CSSD group, with hyperpigmentation surrounding the scar (Fig. 4). Three months later, the local allergic reaction was resolved. No infection, periprosthetic fracture, dislocation, or aseptic loosening occurred. The complete details are shown in Table 2.

**Table 2:** Details of clinical outcomes.

	non-CSSD (N = 52)	CSSD (N = 52)	*P* value
Follow-up (mo)	41.2 ± 7.1	40.5 ± 6.2	.56
*Primary outcome*			
Intraoperative estimated blood loss (mL)	244.5 ± 106.8	229.6 ± 115.2	**.34**
Calculated total blood loss (mL)	508.5 ± 280.3	742.1 ± 330.3	**<.001**
Transfusion amount (unit)	0.03 (0–2)	0.3 (0-4)	**.02**
Patients transfused rates (%)	1 (1.9 %)	9 (17.3 %)	**.02**
Hgb level (g/dL)*	(N = 51)	(N = 43)	
Immediate postoperative	11.6 ± 0.8	11.6 ± 0.7	.97
postoperative d 1	10.7 ± 1.1	10.3 ± 1.0	**.04**
postoperative d 3	10.2 ± 1.1	9.8 ± 0.7	**.03**
Change in Hgb (g/dL)*	(N = 51)	(N = 43)	
From pre to immediate postoperative	1.6 ± 1.1	1.3 ± 1.0	.14
From immediate to postoperative d 3	1.6 ± 1.0	2.0 ± 0.8	**.03**
Length of hospital stay (d)	7.2 ± 1.4	7.8 ± 1.6	**.03**
Total estimated cost (dollars)	162.1 ± 19.4	288.5 ± 82.4	**<.001**
Complication			
Allergic reaction	1	0	.32
*Secondary outcome*			
Operation time (min)	62.3 ± 10.9	60.3 ± 11.2	.35
Closure time (min)	14.2 ± 1.2	12.8 ± 1.4	**<.001**
Time to using crutches following THA (d)	1.8 ± 0.8	2.6 ± 1.4	**.002**
HHS (preoperative)	45.8 ± 9.6	44.8 ± 12.0	.65
HHS (postoperative 1 mo)	84.4 ± 2.1	84.3 ± 2.2	.78
HHS (postoperative 3 mo)	93.4 ± 1.8	92.7 ± 2.2	.10
HHS (at the last follow-up)	97.5 ± 1.2	97.1 ± 1.3	.09

CSSD = closed suction surgical drainage system, HHS = Harris hip score, Hgb = hemoglobin, THA = total hip arthroplasty.

* These values were compared only in the group that did not receive a transfusion.

## 4. Discussion

THA is a widely performed procedure that produces excellent results. Nowadays, suctioning is considered one of the routine techniques in hip replacement. Theoretically, the application of closed suction could promote wound healing and evacuate developing hematomas around surgical wounds. However, a few studies have challenged the superiority of the widespread use of CSSD after total joint replacement.

Several studies have concluded against drain placement after finding no significant differences between the presence or absence of drainage in terms of blood loss, infection, and wound healing. The closed suction system does not reduce the incidence of hematoma and infection. In a prospective observational study, 120 patients with total knee arthroplasty (TKA) were randomly assigned even and odd numbers and divided into 2 groups.^[13]^ They found significant Hgb drops postoperatively in the drain group with an increased number of transfusions than those in the nondrainage group. Suarez et al reported no clinical benefit or disadvantage to closed suction drainage in anterior hip arthroplasty with the concomitant use of tranexamic acid for surgical hemostasis and aspirin for venous thromboembolism prophylaxis.^[1]^ Kumar et al had discouraged the placement of a drain since it does not reduce the incidence of wound complications and postoperative rehabilitation, and doing so also helps to cut expense rates.^[14]^ Moreover, a meta-analysis showed that the routine use of closed suction drainage for elective THA might cause more harm than benefit.^[15]^ The risk of skin microorganisms entering the wound either by the drain or drain track also increased the rate of wound sepsis.^[3]^

In contrast, other investigators support the application of closed suction after orthopedic procedures. Kim et al recommended the routine use of suction drains for wounds after primary THA to reduce drainage, the amount of soaked dressings requiring reinforcement, and the incidence of ecchymosis and erythema.^[16]^ It may also cause a positive psychological impact by reducing the patient fear of bleeding. In their study, nondrained wounds had a higher incidence of deep, large hematomas (54.2%) than drained wounds upon ultrasound examination.

Our results were consistent with those obtained from recent literature on the application of drains in patients with THA.^[1,13–15]^ In our study, we found that the need for blood transfusion in the non-CSSD group is less than that in the CSSD group, which reduces the risk of complications related to blood transfusion. In addition, the changes in Hgb levels in the non-CSSD group were statistically lesser than those in the CSSD group, and we believe that the effect of a tamponade might decrease cutting the medulla and soft tissue bleeding in the non-CSSD group. Our study showed a more prolonged length of hospital stay than other studies.^[2,17]^ Korean governmental medical insurance policy results in a longer hospital stay for patients with THA, since postoperative rehabilitation is also begun.

Skin adhesives have been widely used for wound closure. There are several benefits of skin adhesives, such as easy use, painless application, and excellent cosmetic results. They are also watertight and do not cause needlestick injuries compared with staples or sutures. Additionally, closure with skin adhesive allows patients to shower sooner after surgery if directed by their health care professionals.^[18]^ Previous studies have shown that skin adhesive has significant efficacy in the closure of low-tension lacerations and incisions.^[19–21]^ A recent randomized, prospective, and controlled single-site study by Eggers et al showed that tissue adhesives are an efficient and viable alternative for TKA closure.^[22]^ Khurana et al found that with the help of skin adhesive, the wound will not be disturbed, nor will it be repeatedly exposed to the ward environment due to dressing changes.^[8]^ In addition, the use of skin adhesive can assist in reducing postoperative wound drainage after TKA surgery, which is an excellent supplement.^[6]^

The major drawbacks of this technology include its high price, possible foreign body reactions, and an increased time spent in the operating room. In our study, the overall costs expended by the non-CSSD group were significantly lower than those expended by the CSSD group (162.1 US dollars compared with 288.5 US dollars, *P* < .001). In theory, the price of skin adhesive is much higher than that of the Steri-Strips skin closures. However, the cost of the daily wound dressing, nursing care fees, the closed suction system, and the transfusion associated with drainage make CSSD more expensive overall. Furthermore, the time to wound closure was statistically longer in the non-CSSD group following THA in our study. In the non-CSSD group, the average closure wound time was 1.4 minutes longer than that in the CSSD group for hip replacement. We believe that this increase is acceptable and will not have a severe impact on total operation time. In the present study, there was only 1 patient who experiences an allergic skin reaction with hyperpigmentation due to skin adhesive after surgery, observed in the non-CSSD group. The patient presented with a hyperpigmentation reaction to skin adhesive, which looked like pigmented contact dermatitis, but no signs of dermatitis were observed. The patient had no history of allergy to cosmetic glue. The local allergic reaction resolved after 3 months. Thus, to avoid side effects like allergic skin reactions, skin adhesive should not be used for patients with known or suspected allergies to cyanoacrylate or formaldehyde.

The outcome of the functional assessments in previous studies showed that there was no statistical difference in the mean HHS between the drainage and nondrainage groups.^[16,23,24]^ In this study, we found the same trend in HHSs between both groups. However, the patients in the non-CSSD group could walk earlier following THA than those in the CSSD group. We believe this result is associated with the application of the skin tissue adhesives and the absence of CSSD, which made patients feel more confident in mobilizing the area. Our analysis indicated that non- CSSD with skin adhesive could lead to a more rapid recovery and return to normal functional activity than standard suture methods.

There are several limitations to the present study. First, the study was not a prospective, randomized, and controlled comparison between both groups. Secondly, the types of implants were not uniform since we chose these prostheses to fit the patients’ varying proximal femoral bone geometries. This situation is hard to avoid since the patients had different kinds of diagnoses. The other limitation is that we concentrated on postoperative complications, duration of hospital stay, functional outcomes, and total estimated cost, despite having 2 variables (skin closure and drainage) in our study. We must point out that these need to be further studied.

## 5. Conclusion

Our study demonstrates that the use of skin adhesive without CSSD could help decrease blood loss, the need for transfusion, and the length of hospital stay, and seems to more cost-effectiveness than using CSSD. It may also provide superior results and allow the patient to recover faster. Using this type of skin adhesive without CSSD is an efficient wound closure method for patients undergoing uncomplicated THA. However, care must be taken for allergic reactions, especially for patients with known or suspected allergies to cyanoacrylate or formaldehyde.

## Author contributions

**Conceptualization:** Taek-Rim Yoon, Kyung-Soon Park.

**Data curation:** Chan Young Lee, Sheng-Yu Jin, Hwiwoo Jeong.

**Formal analysis:** Chan Young Lee, Sheng-Yu Jin, Kyung-Soon Park.

**Methodology:** Chan Young Lee, Sheng-Yu Jin, Hwiwoo Jeong.

**Supervision:** Taek-Rim Yoon, Kyung-Soon Park.

**Writing – original draft:** Chan Young Lee, Sheng-Yu Jin.

**Writing – review & editing:** Kyung-Soon Park.
